# Diode Laser Surgery of Recurrent White Lesion of the Lip: Clinicopathological Consideration and Cosmetic Outcome

**DOI:** 10.7759/cureus.7585

**Published:** 2020-04-08

**Authors:** Domenico De Falco, Daniela Di Venere, Eugenio Maiorano

**Affiliations:** 1 Dentistry, University of Bari Aldo Moro, Bari, ITA; 2 Department of Emergency and Organ Transplantation, University of Bari Aldo Moro, Bari, ITA

**Keywords:** diode laser, leukoplakia, oral surgery

## Abstract

Conventional surgery for potentially malignant lesions of the lip may result in unaesthetic sequela. Diode laser allows a careful surgical excision of lip lesions without intraoperative bleeding and stitches, promoting a fast mucosal healing, thereby leading to a complete restitutio ad integrum of lip mucosa without aesthetic complications.

## Introduction

With a worldwide prevalence of 4.4%, oral potentially malignant disorders (OPMDs) are relatively common tissue changes that may precede squamous cell carcinoma, the most common malignancy of the oral mucosa accounting for the 80% to 90% of all oral cancers [1,2]. The most common OPMDs are leukoplakia, speckled leukoplakia, erythroplakia, and actinic cheilitis [3,4]. Leukoplakia is defined by the World Health Organization as "a white patch or plaque that cannot be characterized clinically or pathologically as any other disease" [2]. Leukoplakias occurring on the lower lip as well as on the floor of the mouth and lateral tongue may show more epithelial dysplasia or malignant transformation [3,5]. Overall, persistent and/or recurrent white lesions of the oral mucosa create a suspicion of malignancy and usually require incisional biopsy or complete excision [2,4,5].

We report a case of recurrent white lesion of the lower lip treated with laser diode excision for an optimal aesthetic outcome.

## Case presentation

**Figure 1:**
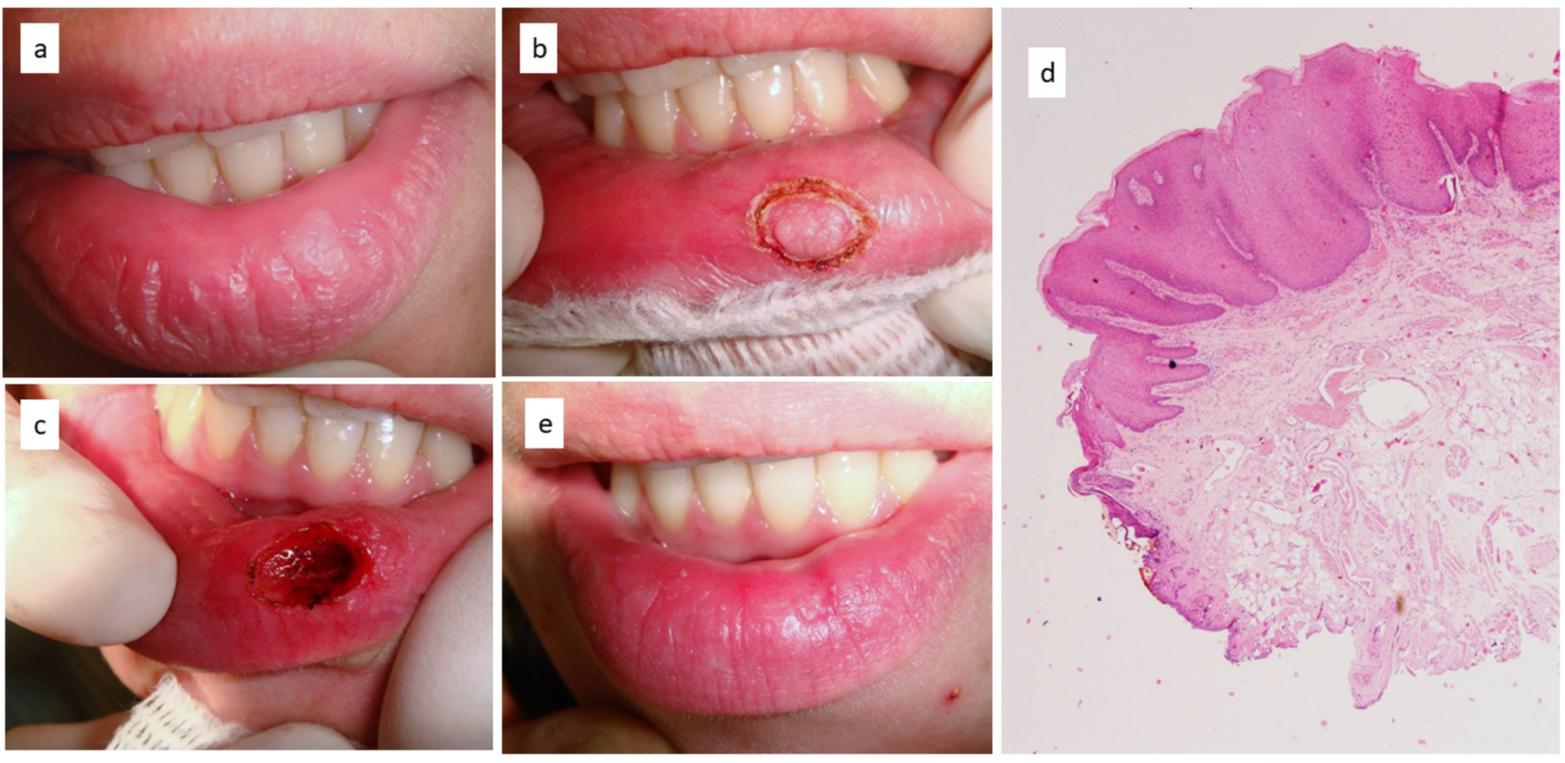
Recurrent white lesion of lip vermillion (a); preliminary definition of safe margins of excision by diode laser (b); deep surgical wound immediately after laser excision (c); histological examination leading to the diagnosis of frictional keratosis (H&E, X2) (d); complete mucosal healing 14 days later without cosmetic complications (e)

A 32-year-old woman was referred for management of a recurrent lesion of the lower lip. Clinical examination revealed a white plaque (Figure 1a). The lesion was described by the patient as chronic, and it had recurred despite having undergone treatment twice previously. No other white lesions were detectable in the oral cavity. Diode laser excision of the entire lesion followed by histological examination was suggested, and the patient agreed. Under light conscious sedation to reduce dental anxiety and with local infiltration of anesthesia, the lesion was removed with wide lateral and deep margins using a diode laser with a wavelength of 980 ± 10 nm in continuous wave, an output energy of 1 W, and a fiber of 320 microns (Figures 1b, 1c). Bleeding was absent during the entire procedure, and no stitches were necessary. The tissue was sent for a histological examination leading to the diagnosis of friction keratosis. No morphological alterations related to the thermal cut of the diode laser were observed (Figure 1d). The lip mucosa showed complete healing after 14 days without cosmetic complications (Figure 1e).

## Discussion

Although rare, several lesions such as actinic keratosis, leukoplakia, carcinoma in situ, and superficially invasive carcinomas may occur in the lower lip [2,3,5]. Current treatment modalities include cryosurgery or electrosurgery, topical chemotherapy, or lip shave, but all have limitations including minor deformation of the lip [6].

The diode laser, potassium titanyl phosphate (KTP), carbon dioxide (CO2), neodimio:YAG (yttrium aluminum garnet), and erbium:YAG are medical devices widely used in oral surgery [7,8]. Nevertheless, among all lasers with surgical capabilities, especially contextual cut and coagulation, the diode laser is surely the most commonly used for excision of benign and malignant proliferating lesions of the oral cavity, photocoagulation of both small and large vascular malformations of the head and neck, and adjunctive periodontal therapy [9-12].

The advantages of diode laser are the absence of intraoperative bleeding, reduction of postoperative edema, unnecessary stitches, and fast mucosal healing by second intention, avoiding unaesthetic complications for lesions occurring in the lip vermilio [13,14].

As in the reported case, white lesions of the lip surely need decisive surgical intervention, especially when persistent and/or recurrent and when an incisional biopsy cannot be performed [3,5]. Excision should have safe lateral margins and adequate depth, while avoiding cosmetic complications as much as possible. The diode laser used in the current case allows for careful and decisive excision of the lesion without thermal damage to the surrounding tissues, with an absence of bleeding and unnecessary stitches, resulting in faster healing of the surgical wound and a pleasing cosmetic outcome [13,14].

Diode laser surgery can simplify oral surgery procedures, especially in children. It is also useful in patients with contagious infectious diseases (such as hepatitis C or B viruses and HIV) as it minimizes the risk of bleeding and potential-related infectious concerns during perioperative care [15,16]. In the case of dental anxiety, conscious sedation is suggested as it can improve the acceptability of the treatment [17,18].

## Conclusions

The diode laser represents a safe and decisive tool for surgical and nonsurgical treatments in the oral cavity. For white lesions occurring in the lip, diode laser surgery allows for meticulous treatment and helps prevent cosmetic complications while providing effective and safe recovery.
